# Impact of reirradiation, chemotherapy, and immunotherapy on survival of patients with recurrent lung cancer: A single‐center retrospective analysis

**DOI:** 10.1111/1759-7714.13884

**Published:** 2021-02-14

**Authors:** Brane Grambozov, Romana Wass, Markus Stana, Sabine Gerum, Josef Karner, Gerd Fastner, Michael Studnicka, Felix Sedlmayer, Franz Zehentmayr

**Affiliations:** ^1^ Department of Radiation Oncology Paracelsus Medical University, SALK Salzburg Austria; ^2^ Department of Pneumology Paracelsus Medical University, SALK Salzburg Austria; ^3^ Department of Pulmonology Kepler University Hospital Linz Austria; ^4^ radART – Institute for Research and Development on Advanced Radiation Technologies Paracelsus Medical University Salzburg Austria

**Keywords:** immunotherapy, local recurrence, lung cancer, overall survival and toxicity, reirradiation

## Abstract

**Background:**

Given the limited curative treatment options for recurrent lung cancer patients, the aim of our retrospective study was to investigate whether these patients would benefit in terms of overall survival (OS) by adding immunotherapy to high‐dose reirradiation.

**Materials and methods:**

Between 2013 and 2019, 47 consecutive patients with in‐field tumor recurrence underwent high‐dose thoracic reirradiation at our institute. Twenty patients (43%) received high‐dose reirradiation only, while 27/47 (57%) additionally had systemic therapy (immunotherapy and/or chemotherapy). With the exception of one patent, the interval between first and second radiation was at least 9 months. All patients had an Eastern cooperative oncology group ≤2. The diagnostic work‐up included a mandatory fluorodeoxyglucose‐positron emission tomography‐computed tomography scan and histological verification. The primary endpoint was OS after completion of the second course of irradiation.

**Results:**

In the whole cohort of 47 patients, the median overall survival (mOS) after reirradiation was 18.9 months (95% confidence interval [CI] 16.5–21.3 months), while in the subgroup of 27 patients who received additional systemic treatment after reirradiation, mOS amounted to 21.8 months (95% CI 17.8–25.8 months). Within this group the comparison between reirradiation combined with either immunotherapy (*n* = 21) or chemotherapy (*n* = 6) revealed a difference in OS, which was in favor of the first (log‐rank *p* value = 0.063). Three patients (11%) experienced acute side effects and one (4%) showed a late hemorrhage grade 3.

**Conclusion:**

Patients who received immunotherapy and reirradiation lived longer than those who did not receive immunotherapy.

## Graphical abstract

The aim of our retrospective study was to investigate whether reccurrent lung patients would benefit in terms of overall survival by adding immunotherapy to high‐dose reirradiation. Between 2013 and 2019, 47 consecutive patients with in‐field tumor recurrence underwent high‐dose thoracic reirradiation at our institute. Twenty patients (43%) received high dose reirradiation only, while 27/47 (57%) additionally had systemic therapy (immunotherapy and/or chemotherapy). Patients who received immunotherapy and reirradiation lived longer than those who did not receive immunotherapy.

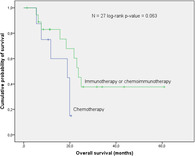

**Abbreviations**
CCI: Charlson co‐morbidity index
CTCAE: common toxicity criteria for adverse events
DART: dose‐differentiated accelerated radiotherapy
ECOG: Eastern cooperative oncology group
EQD2: biologically equivalent dose in 2 Gy fractions
FDG‐PET‐CT: fluorodeoxyglucose‐positron emission tomography
IMRT: intensity modulated radiotherapy
mOS: median overall survival
NSCLC: nonsmall cell lung cancer
OS: overall survival
RT: radiotherapy
SABR: stereotactic ablative body therapy
SCLC: small‐cell lung cancer
TNM: tumor, node, metastasis
VMAT: volumetric intensity modulated arc therapy

## INTRODUCTION

Immunotherapy has led to revolutionary advancements in cancer treatment^1^, ^2^, ^3^, ^4^, ^5^, ^6^, ^7^, ^8^ and has given new hope to the large number of patients who die from lung cancer each year.^9^ Immunotherapy is now a standard first‐ and second‐line therapy for patients with advanced lung cancer.^1^, ^2^, ^3^, ^4^, ^6^, ^8^

With the aim of improving the options available for the treatment of lung cancer patients and based on the potential synergistic effect of radiation therapy and immunotherapy in terms of both local and systemic antitumor response as already described in preclinical models,^10^, ^11^, ^12^ the interest of the oncological community in combining these therapeutic modalities in a clinical setting has increased.^1^, ^2^, ^13^, ^14^ As a result, clinical studies have shown remarkable benefits in terms of both progression‐free and overall survival (OS) in lung cancer patients with acceptable toxicity,^1^, ^2^, ^13^ which may be attributed to the synergistic antitumor effect mentioned above. In contrast, numerous publications have suggested that the very complex interaction between the irradiated cells, tissue, and the immune system could enhance the effect of immunotherapy.^1^, ^2^, ^5^, ^7^, ^12^, ^15^, ^16^, ^17^

In light of this, the potential for antitumor immune activation by irradiation could play an important role for radiotherapy in systemic disease, especially since the presence of immunosuppressive mediators in the tumor microenvironment could limit the number of patients who experience the therapeutic benefits of immunotherapy.^12^, ^18^ Despite progress in all related clinical disciplines, there is a need to improve clinical outcomes in patients at all tumor, node, metastasis (TNM) stages, including those with recurrent lung cancer for whom curative treatment is already limited.^5^, ^19^, ^20^ Hence, combining therapeutic modalities such as radiotherapy, chemotherapy, and immunotherapy in the hope of overcoming their therapeutic limitations and achieving a synergistic effect seems plausible.^1^, ^2^ With respect to the combination of reirradiation for loco‐regional relapse and immunotherapy, there is currently one review extant.^5^ Additionally, the results of an ongoing study are pending (ClinicalTrials.gov identifier NCT03087760), which—as opposed to our study—uses proton reirradiation rather than photons.

The aim of our retrospective analysis was to investigate whether patients with recurrent lung cancer would benefit from adding immunotherapy to ablative reirradiation in terms of OS, taking into account toxicity. Local control after reirradiation and dose to organs at risk are the subject of another paper that is currently under review.

## METHODS

### Patients

Between 2013 and 2019, 47 consecutive patients who underwent high‐dose thoracic reirradiation were included in a prospective observational database. While 20 patients were reirradiated only, 27 received systemic therapy in addition to high‐dose reirradiation. In this subgroup, immunotherapy was administered alone or with chemotherapy in 21/27 patients (78%). Six out of 27 patients (22%) received chemotherapy alone. The inclusion criteria were as follows: (1) all patients had to be classified as inoperable and in all patients both the primary and the secondary tumor had to be located in the lungs; (2) if possible, patients should have received two courses of curatively intended radiation therapy with a time interval of 9 months or more between them (an exception was made in only one patient who was reirradiated 5 months after the first radiation therapy treatment); (3) the tumor was histologically verified and categorized according to the 8th edition of the TNM classification; (4) fluorodeoxyglucose‐positron emission tomography‐computed tomography (^18^F‐FDG‐PET‐CT) was required in the diagnostic work‐up; (5) the performance status had to be ≤2 according to the Eastern Cooperative Oncology Group (ECOG). Patients who received palliative radiation treatment, postoperative radiotherapy (RT), or those with chest wall tumors and/or out‐of‐field tumor recurrences were excluded. All patients were discussed in a multidisciplinary tumor board with pneumologists, medical oncologists, radiologists, thoracic surgeons, pathologists, and radiation oncologists. This study was reviewed and approved by the ethics committee of the Federal Province of Salzburg (No. 1070/2020).

### Radiation and systemic therapy

Patients were reirradiated using intensity modulated radiotherapy (IMRT/VMAT) or stereotactic body radiotherapy (SABR). A planning computed tomography (CT) scan with an acquisition time of 3 s was performed prior to IMRT/VMAT. Additionally, four‐dimensional computed tomography (4D‐CT) was performed in SABR patients. Patients were immobilized using a vacuum cradle and WingSTEP. Subsequently, the planning CT was registered with ^18^F‐FDG‐PET‐CT. For SABR patients, the internal target volume (ITV) was created by contouring the gross tumor volume (GTV) on three breathing phases (expiration, inspiration, and average) and their subsequent union (ITV = CTV‐clinical target volume). The planning target volume (PTV) was created by adding a symmetric margin of 5 mm to the ITV and an additional 4 mm margin in the cranio‐caudal direction. In IMRT/VMAT patients, the GTV was contoured on a so called “slow CT” with an acquisition time of 4 s. This GTV actually constitutes an ITV/CTV as it includes the respiration‐dependent movement of the tumor. The PTV was defined by adding a symmetric margin of 7 mm to GTV. IMRT/VMAT was delivered in three fractionation regimens: dose‐differentiated accelerated RT in twice daily fractions of 1,8 Gy (dose‐differentiated accelerated radiotherapy [DART]‐bid) as described in two previous publications,^21^, ^22^ conventionally with 2 Gy per fraction, and hypofractionated RT (one fraction of 3 Gy per day). SABR included two different schemes: eight fractions of 8 Gy (65% isodose) delivered daily for central tumors (i.e. within 2 cm of the proximal bronchial tree) and three fractions of 15.4 Gy in (65% isodose) every other day for peripheral tumors. Since various fractionation regimens were used, total radiation doses were compared by biologically equivalent dose in 2 Gy fractions (EQD2). Organs at risk (OAR), such as esophagus, central vessels and airways, spinal cord, lungs, and heart were routinely contoured and dose volume histograms of both initial and reirradiation plans were used to determine the cumulative radiation dose of each critical organ.

Prior to reirradiation, patients with nonsmall cell lung cancer (NSCLC) received two cycles of either cisplatin (75 mg/m^2^/d) combined with pemetrexed (500 mg/m^2^/d) or gemcitabine (1000 mg/m^2^/d), while small‐cell lung cancer (SCLC) patients received four cycles of cisplatin (75 mg/m^2^/d) together with etoposide (120 mg/m^2^ days 1 to 3). In the case of renal dysfunction carboplatin at an area under the curve (AUC) of 5 on day 1 (absolute maximum dose 1100 mg) was applied instead of cisplatin. Depending on the tumor histology, patients received one of the following immunotherapeutic agents after the second ablative radiation therapy: atezolizumab, durvalumab, nivolumab, or pembrolizumab.

### Toxicity

The Common Terminology Criteria for Adverse Events version (CTCAE) 5.0 were used to report toxicity. Grade 1 toxicities were not considered as clinically relevant and have therefore not been assessed in this study. A cutoff of 90 days after completion of reirradiation was used to distinguish between acute and late toxicities, with the exception of pneumonitis, which was still considered acute if it occurred within 180 days of the end of RT.

### Follow up

Patients were seen 6 weeks after completion of radiotherapy, then every 3 months for the first 2 years and twice a year thereafter. Clinical examinations, contrast‐enhanced CTs, and pulmonary function tests were performed at every follow‐up. If local recurrences or new lung lesions were suspected on the chest CT, ^18^F‐FDG‐PET‐CT was performed. Local relapse was defined as tumor growth within the reirradiated volume covered by the 95% or 65% isodose after IMRT/VMAT or SABR, respectively.

### Statistics

The primary endpoint was OS, which was calculated using the Kaplan–Meier method. We defined OS as the time between the end of reirradiation and death or latest follow‐up. Although the subgroup of 27 patients was of interest for our analysis, a total of 47 patients—20 of whom were only reirradiated—were also analyzed. With the aim of retaining as much potential information regarding the effects of the three therapy modalities on OS as possible, the threshold for first‐order errors (*α*) was set at 0.2, which is a more permissible limit usually used in exploratory studies.^23^, ^24^ For intergroup comparisons the log‐rank test was used.

## RESULTS

### Patients

Of the 47 patients in the whole cohort, 29 (62%) were men and 18 (38%) were women. The median age at the start of the reirradiation was 66 years (range 52–83 years) in both the entire cohort and the subgroup. Based on histological findings at initial diagnosis, 35/47 (75%) patients had NSCLC and 10/47 (21%) patients had SCLC across the cohort. No pathological confirmation could be obtained in two patients (4%). For details, see Table 1.

**TABLE 1:** Patient‐ and treatment‐related parameters in the cohort (*N* = 47)

Patients *N* = 47			
Patient parameters	Age (years)	Median	66,3
		Range	52–83
	Sex	Male	29
		Female	18
	Weight loss (%)	>5%	22
		<5%	25
	ECOG	0–1	40
		2	7
	Histology	SCLC	10
		NSCLC	35
		Unknown	2
	T stage	*x*	3
		1	9
		2	21
		3	9
		4	5
	N stage	0	11
		1	7
		2	22
		3	7
	M stage	0	40
		1	7
	UICC stage	I	7
		II	8
		III	25
		IV	7
	FEV1 (%)	Median	71
		Range	35–100
	COPD grade	0	17
		1	3
		2	9
		3	10
		4	6
		Unknown	2
	Charison Comorbidity Index	Median	5
		Range	2‐10
Treatment‐related parameters	Reirradiation volume (ml)	Median	47
		Range	4–541
	Tumor location (*n*)	Peripheral	22
		Central	25
	Cumulative EQD2 (Gy)	Median	131
		Range	77‐339
	Systemic therapy (*n*)	Yes	27
		No	20
	Interval between radiation courses (months)	Median	20
		Range	5–145
	Radiation technique	Accelerated	23
		STX	13
		Conventional (= 2 Gy/d)	6
		Hypofractionated	5

*Note*: Tx‐means that the tumor was not able to be evaluated

Abbreviations: COPD, chronic obstructive pulmonary disease; ECOG, Eastern cooperative oncology group; EQD2, biologically equivalent dose in 2 Gy fractions; FEV1, forced expiratory volume during the first second; N stage, lymph nodes; NSCLC, nonsmall cell lung cancer; M stage, metastasis; SCLC, small cell lung cancer; STX, stereotactic body irradiation; T stage, tumor; UICC, Union for International Cancer Control.

The subgroup included 27 patients, of whom 17 (63%) were men and 10 (37%) were women. All tumors were histologically verified at initial diagnosis, according to which 21/27 patients (78%) had NSCLC and 6/27 patients (22%) had SCLC. The vast majority of patients (25/27, 92.5%) had an ECOG performance score ≤1 with a mean Charlson co‐morbidity index (CCI) of 6 (range 3–10). More than half of the patients (16/27, 60%) had stage III disease. Four patients (15%) were classified as oligometastic at reirradiation. Further details are shown in Table 2.

**TABLE 2:** Patient‐ and treatment‐related parameters in the systemic therapy subgroup (*N* = 27)

Patients *N* = 27			
Patient parameters	Age (years)	Median	66,3
		Range	52–83
	Sex	Male	17
		Female	10
	Weightless (%)	>5%	13
		<5%	14
	ECOG	0–1	25
		2	2
	Histology	SCLC	6
		NSCLC	21
	T stage	*x*	2
		1	6
		2	11
		3	4
		4	4
	N stage	0	5
		1	4
		2	13
		3	5
	M stage	0	23
		1	4
	UICC stage	I	2
		II	5
		III	16
		IV	4
	FEV1 (%)	Median	71,1
		Range	36–100
	COPD grade	0	13
		1	1
		2	4
		3	6
		4	2
		Unknown	1
	Charison comorbidity index	Median	6
		Range	3–10
Treatment‐related parameters *N* = 27	Reirradiation volume (ml)	Median	48.8
		Range	4.5–217
	Tumor location (*n*)	Peripheral	14
		Central	13
	Cumulative EQD2 (Gy)	Median	132,8
		Range	79–211
	Systemic therapy	Chemotherapy	6
		Immunotherapy with/without Chemotherapy	21
	Interval between radiation courses (months)	Median	14
		Range	5–80
	Radiation technique	Accelerated	13
		STX	6
		Conventional (= 2 Gy/d)	5
		Hypofractionated	3

Abbreviations: COPD,chronic obstructive pulmonary disease; ECOG, Eastern cooperative oncology group; EQD2, biologically equivalent dose in 2 Gy fractions; FEV1, forced expiratory volume during the first second; N stage, lymph nodes; NSCLC, nonsmall cell lung cancer; M stage, metastasis; SCLC, small cell lung cancer; STX, stereotactic body irradiation; T stage, tumor; UICC, Union for International Cancer Control.

### Reirradiation and systemic therapy

While 20 of the 47 patients were only reirradiated (43%), 27/47 (57%) received systemic therapy in addition to reirradiation. In this subgroup of interest, the tumor was located peripherally in 14/27 (52%) patients and centrally in 13/27 (48%) patients. Almost half of the patients (13/27, 48%, median EQD2 128 Gy, range 89–150.5 Gy) were reirradiated with DART‐bid, while 6/27 (22%, median EQD2 191 Gy, range 148–211 Gy) received SABR; in 5/27 patients (19%, median EQD2 122 Gy, range 79–134 Gy) conventional radiation therapy was applied and in 3/27 (11%, median EQD2 99 Gy, range 94–135.5 Gy) a hypofractionated schedule was used. The median reirradiation PTV was 48.8 ml (range 4.5–217 ml) and the median cumulative radiation dose EQD2 delivered in both treatments was 132.8 Gy (range 79–211 Gy). The median interval between the first and second treatment courses was 14 months (range 5–80 months). Twenty‐one patients (78%) received immunotherapy with or without chemotherapy (Table 2). The immunotherapeutic agents were administered after reirradiation over a median treatment time of 6 months (range 0.5–24 months). Six patients (22%) received chemotherapy alone prior to reirradiation.

### Overall survival

**FIGURE 1:**
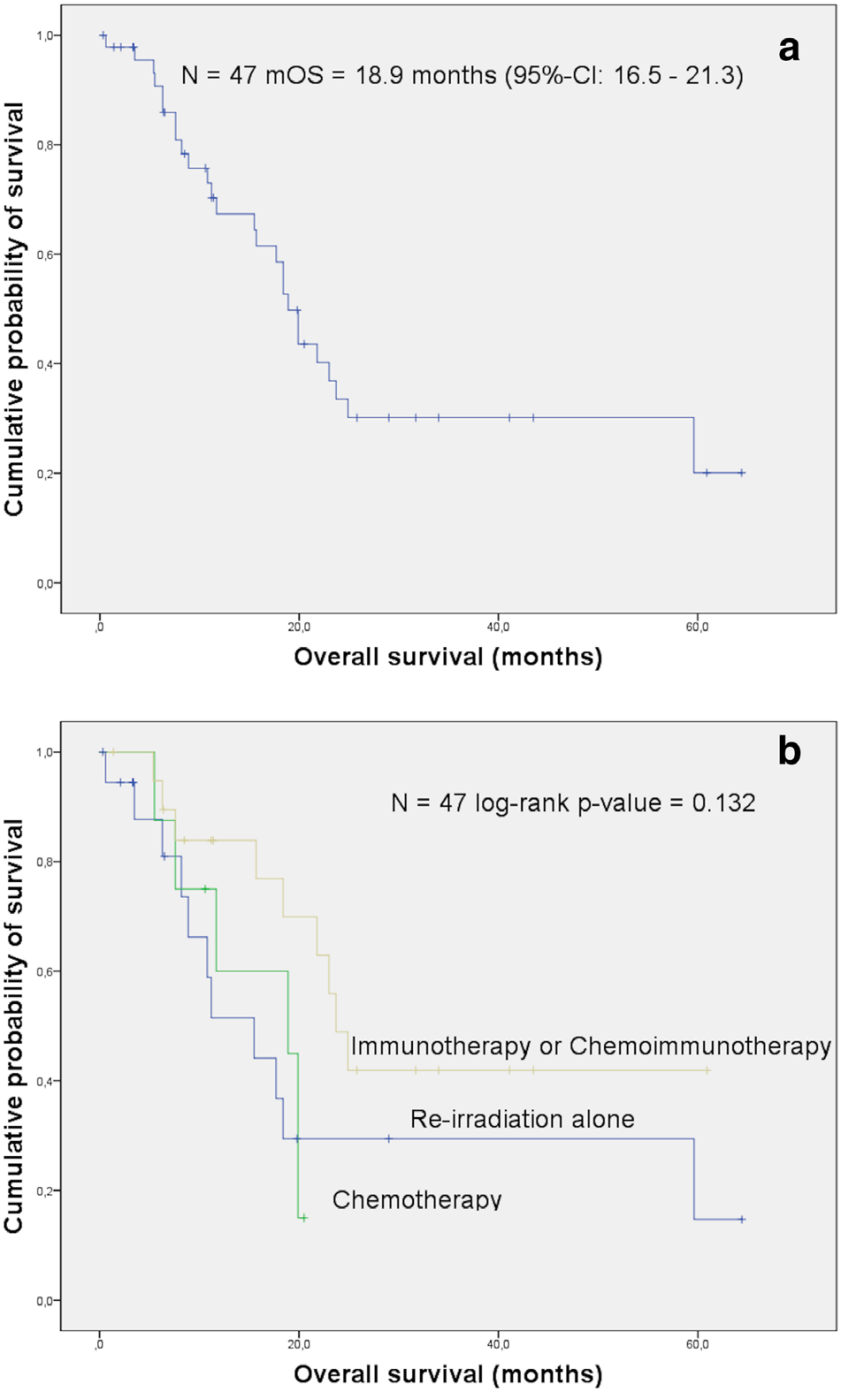
(a) The median overall survival (mOS) in the whole cohort of 47 patients was 18.9 months (95% confidence interval [CI] 16.5–21.3 months). (b) The 47 patients in the whole cohort were stratified according to the type of systemic therapy received together with reirradiation: immunotherapy or immunochemotherapy (orange), reirradiation alone without systemic treatment (blue), chemotherapy (green). Of these, the first group had the longest survival (mOS 23.7 months, 95% CI 20.3–27.1 months, overall log‐rank *p* value = 0.132)

The median follow‐up across the cohort was 11.7 months (range 0.3–64.4 months). Of the 47 patients, 21 are still alive (45%). The median OS (mOS) after reirradiation was 18.9 months (95% CI 16.5–21.3 months; Figure 1(a)). The difference in OS between the three treatment modalities in the whole cohort, i.e. reirradiation only vs. reirradiation plus chemotherapy vs. reirradiation plus immunotherapy with/without chemotherapy was in favor of the third group (log‐rank *p* value = 0.132; Figure 1(b)).

**FIGURE 2:**
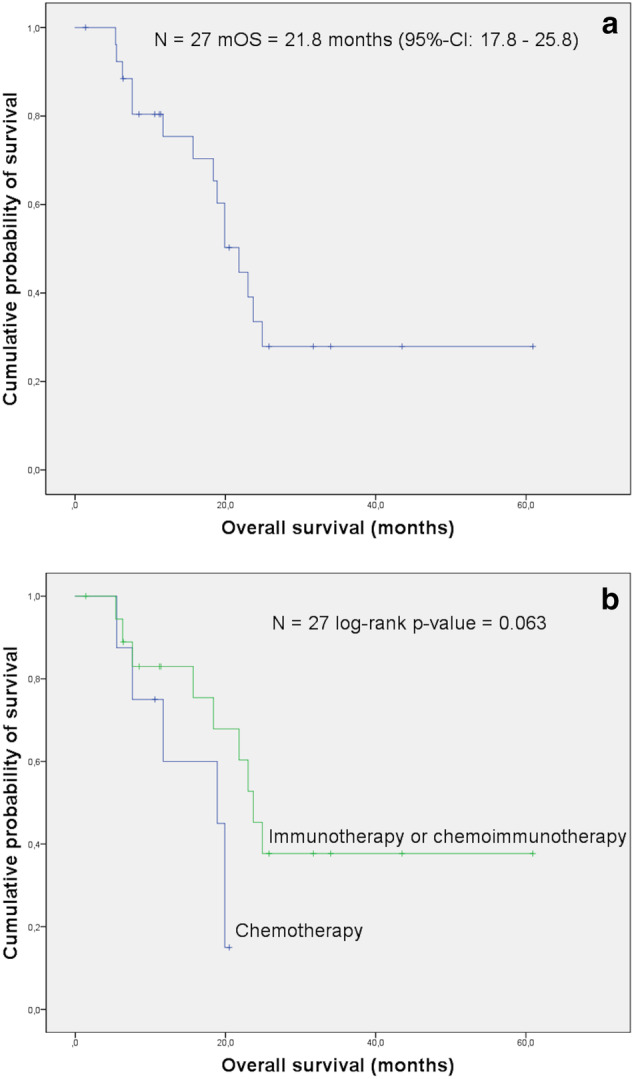
(a) The median OS (mOS) in the systemic therapy subgroup was 21.8 months (95% confidence interval [CI] 17.8–25.8 months). (b) Patients who received immunotherapy or chemo‐immunotherapy together with reirradiation lived longer than patients who underwent reirradiation with chemotherapy alone (mOS 23.7 months, 95% CI 20.3–27.1 months, log‐rank *p* value = 0.063)

In the immunotherapy subgroup, the mOS after the second radiation course was 21.8 months (95% CI 17.8–25.8 months; Figure 2(a)). Patients were followed up for a median of 18.4 months (range 1.4–60.9 months), and of these 12 (44%) are still alive, while 14 (52%) patients died from cancer‐related conditions. One patient (4%) died from peritonitis caused by bacterial infection. The median local progression‐free survival was 7.9 months (95% CI 6.7–9 months). The difference in OS was in favor of the immunotherapy subgroup (log‐rank *p* value = 0.063; Figure 2(b)).

### Toxicity

Of the 47 patients, eight (17%) experienced acute side effects greater than or equal to grade 2 and 1/47 (2%) patients had late ≥grade 2 toxicity. A grade 5 acute heart failure 1 week after the end of reirradiation was reported in 1/47 (2%) patients with no history of cardiac disease. In this patient, the cumulative maximum EQD2 delivered in both radiation courses was 110 Gy, which was below the 115 Gy classified as tolerable in the literature.^25^ The 43% total V20 lung (volume receiving ≥20 Gy) met the above limitation while the 45% V25 (volume receiving ≥25 Gy) heart did not because the tumor was in the central upper lobe including the left hilum and upper segments of the lower lobe. Since a therapeutic cause of death, although unlikely, could not be entirely excluded this patient was scored as having grade 5 toxicity (Table 3).

**TABLE 3:** Treatment‐related toxicity in the whole cohort

Toxicity (*N* = 47)						
Type of toxicity		Grade 1	Grade 2	Grade 3	Grade 4	Grade 5
Acute	Esophagitis	na	4	2	0	0
	Pneumonitis	na	1	0	0	0
	Heart	na	0	0	0	1
Late	Esophagitis	na	0	0	0	0
	Pneumonitis	na	0	0	0	0
	Hemorrhage	na	0	1	0	0
	Chest wall pain	na	0	0	0	0

Abbreviation: na, not assessed.

In contrast, there were no grade 4 or 5 toxicities in the immunotherapy subgroup. Acute toxicities occurred as grade 2 in three patients (11%). Two of these patients had acute esophagitis and one reported acute pneumonitis. A late grade 3 hemorrhage occurred in one patient (4%, Table 4).

**TABLE 4:** Treatment‐related toxicity in the subgroup

Toxicity (*N* = 27)						
Type of toxicity		Grade 1	Grade 2	Grade 3	Grade 4	Grade 5
Acute	Esophagitis	na	2	0	0	0
	Pneumonitis	na	1	0	0	0
	Heart	na	0	0	0	0
Late	Esophagitis	na	0	0	0	0
	Pneumonitis	na	0	0	0	0
	Hemorrhage	na	0	1	0	0
	Chest wall pain	na	0	0	0	0

Abbreviation: na, not assessed.

## DISCUSSION

In this analysis we could show that in patients with locoregional relapse of lung cancer a second course of irradiation together with immunotherapy leads to better OS than a combination with chemotherapy (log‐rank *p* value = 0.063; Figure 2(b)).

Our finding is consistent with a concept published in a review by Evans^5^ intended for recurrent lung cancer patients who already have limited chances of successful curative treatment.^5^, ^19^, ^26^, ^27^ According to Evans,^5^ the combination of the two therapy modalities would have a synergistic effect in terms of both local and systemic disease control, given the high potential for local and systemic failure, possibly due to radiation resistance and the aggressiveness of the disease in recurrent lung cancer patients.^5^ Immunotherapy could potentially play an important role in enhancing the effectiveness of reirradiation and vice versa, which could hypothetically explain the prolonged survival of the subgroup who received reirradiation followed by immunotherapy in our study.

In the absence of published studies on reirradiation with immunotherapy, this consideration relies on data from patients receiving first‐time irradiation, assuming that outcome and toxicity would be similar in the reirradiation setting.^1^, ^2^, ^3^, ^4^, ^5^, ^6^, ^7^, ^8^, ^15^, ^16^, ^20^, ^28^, ^29^, ^30^ In this context, there is data already available on the combination of RT and immunotherapy with primarily curative intent, indicating the potential clinical benefit in lung cancer patients.^1^, ^2^ This could be attributed to the potential synergistic effect of radioimmunotherapy, resulting in a local and systemic anti‐tumor response, which is currently attracting great academic interest and generating many hypotheses about the exact trigger and interaction mechanism behind it.^5^, ^7^, ^12^, ^17^, ^18^, ^29^ In this regard, a recently published review^17^ highlighted the possible synergistic benefits of combining chemotherapy, radiation therapy, and immunotherapy such as the increase of cytotoxicity, the enhancement of immunogenic cell death and tumor necrosis as well as increased tumor‐derived and neoantigen generation, all of which could lead to a potentially enhanced antitumor effect.

The details of the complex mechanism of immunotherapy and radiation, as well as the interaction between the two, are described elsewhere.^5^, ^7^, ^16^, ^17^, ^29^, ^30^ Briefly summarized, tumor cells evade the immune response by up‐regulating specific proteins such as programmed cell death 1 ligand 1 (PDL‐1) on their surface. These immune checkpoint ligands interact with the programmed cell death protein 1 (PD‐1) surface receptors of activated cytotoxic T cells, thereby inhibiting them. By inhibiting the PD‐1/PDL‐1 signaling pathway with inhibitors such as nivolumab, pembrolizumab, durvalumab, and atezolizumab, which were administered in our immunotherapy subgroup, the T cells can recognize the tumor cells as pathogens and eliminate them.^1^, ^2^, ^5^, ^7^, ^20^ Relatedly, radiation‐induced antitumor activity is immune‐mediated by the T cells.^11^ Radiation stimulates tumor antigen presentation on the surface of dendritic cells to T cells, which is to prime the T cells in the lymph nodes to respond effectively against tumor cells.^15^, ^18^, ^31^

The sequence in which RT and immunotherapy would be applied is still under investigation,^13^, ^32^ however available data showed clinical benefit with acceptable toxicity when immunotherapy was administered after radiation treatment,^12^, ^14^ which corresponds to the toxicity results obtained in our study. Accordingly, in our study, considering the side effects of immunotherapy, particularly with regard to pneumonitis^33^ and the severe systemic^7^ and local side effects that could be caused by the reirradiation treatment, immunotherapy was given after reirradiation. This treatment sequence was well tolerated. Eleven percent of the patients experienced grade 2 toxicity, with esophagitis and pneumonitis being the only radiogenic side effects, and 4% reported grade 3 toxicity. No grade 4 or 5 toxicity events were reported.

An obvious weakness of our analysis is the rather permissive threshold for first‐order errors (*α*). However, this is not unusual in exploratory studies with the aim of extracting as much potentially important information as possible.^23^, ^34^ Despite the small cohort and the retrospective nature, our data may gain additional significance given the fact that prospective studies on the combination of reirradiation combined with immunotherapy are lacking.

## CONCLUSION

The combination of reirradiation with immunotherapy could potentially prolong survival with acceptable toxicity. Although prospective studies are warranted, we believe that this combined treatment approach can transform the way patients with recurrent lung cancer are treated.

## CONFLICT OF INTEREST

All authors declare that they have no conflict of interest.
